# Using the jigsaw technique to teach patient safety

**DOI:** 10.1080/10872981.2019.1710325

**Published:** 2019-12-29

**Authors:** Nirvani Goolsarran, Carine E. Hamo, Wei-Hsin Lu

**Affiliations:** Department of Medicine, Stony Brook University Hospital, Stony Brook, NY, USA

**Keywords:** Medical education, patient safety, teaching strategies, medical errors, clinical reasoning

## Abstract

**Background**: Medical education is rapidly changing where there has been decreased emphasis on passive didactics and increased focus on novel modes of teaching and learning to address the unique needs of millennial learners. As educators, it is challenging to keep up and find active teaching strategies outside of routine small group exercises to engage learners. Although the traditional small group activities, such as cased-based learning, allows for interactive and effective teaching, this modality may require the use of multiple faculty facilitators, which can be a difficult resource to find. The jigsaw learning method is cooperative learning that utilizes peer teaching and promotes collaborative learning, and additionally, only one facilitator is required of this type of learning technique.

**Objectives:** We aimed to assess the effectiveness of the jigsaw method by comparing it to the traditional small group learning method to teach principles of diagnostic reasoning. **Design:** Residents were assigned to either the traditional small group teaching method or the jigsaw method. We compared pre-test, post-test, one-year follow-up test results between participants, and resident perception of the exercises.

**Results**: A 2 × 3 repeated measures ANOVA indicated statistically significant improvement in tests scores from before to after participation with the jigsaw method compared to the traditional small group method. Post-survey demonstrated higher resident satisfaction with the jigsaw method.

**Conclusion**: Our study demonstrates that a jigsaw cooperative learning approach can be used as an effective method to promote collaborative learning and engagement.

## Introduction

Current medical education literature emphasizes the use of active learning and teamwork as an effective method to teach and engage learners. One of the barriers to teaching in an active and interactive manner is the lack of resources including available faculty members who are willing and able to facilitate and teach. It is also often challenging for educators to find innovative strategies to promote diagnostic reasoning. Furthermore, as medical education continues to evolve, there has been decreased emphasis on passive classroom didactics and increased focus on active modes of learning to address the unique needs of millennial learners. Although the traditional small group activities, such as case-based and problem-based learning, allow for interactive and effective teaching, these modalities may require the use of multiple faculty facilitators, which can be a difficult resource to recruit due to competing priorities and limited faculty availability of busy clinicians.

The jigsaw method has been employed since the 1970’s and has proven to be effective in high school and college education [1]. This is a strategy in which members of the class are organized into groups and then rearranged into new groups to share their learning through peer teaching [1]. Students in the expert groups are expected to master the materials assigned to them and discuss how to best peer teach the material. When the discussions within the expert groups are complete, one student from each expert group joins a predetermined jigsaw group. The members of the jigsaw group teach each other what they have learned from the assigned materials [2]. A single faculty facilitator oversees the group discussions to ensure the groups stay on task and clarify any misconceptions the students may have. Problem solving and critical thinking skills are fostered during the group discussions where students develop an understanding of the material not possible if they were to learn on their own. This cooperative learning approach enable peers to work within their team in an interdependent manner whereby each individual is held accountable for the content and peer teaching [1].

While the jigsaw method has been employed and proven to be effective in various settings [2–4], to our knowledge, it has not been used in medical education to teach resident trainees. There has been limited information on using this method in topics related to medical education. We aimed to assess the effectiveness of the jigsaw method by comparing it to the traditional small group learning method to teach principles of diagnostic reasoning. Although the jigsaw method can be applicable to many topics in medical education, we chose to apply this learning technique to our diagnostic reasoning lecture series since this topic highlights problem solving techniques.

## Methods

Stony Brook University Hospital is an academic medical center and serves as the tertiary care center of Suffolk County, New York. The internal medicine residency program consists of approximately 96 trainees from Post-Graduate Year (PGY) one through three.

### Participants

During the months of January to March 2017, we conducted a 2-hour interactive patient safety workshops at Stony Brook University Hospital. Each workshop was conducted five times to capture a total of 51 Post Graduate Year (PGY) one and two Internal Medicine residents participated in the study.

### Program description

Participants were randomly assigned to either the traditional small group teaching method (N = 20) or the jigsaw method group (N = 31). Each workshop involved 10–12 residents and each group consisted of 3–4 residents. The Institutional Review Board at Stony Brook University determined this to be an exempt study.

#### Study flow

Each patient safety workshop session began with a one page written morbidity script depicting a diagnostic error [5]. After each individual read the morbidity script independently, all participants were randomly split into either one of two groups: the jigsaw method intervention group (hereafter referred to as the jigsaw intervention group) or the traditional small group teaching method group (hereafter referred to the as the traditional small group). Both groups were further broken down into smaller subgroups of 3–4 residents each, and they were tasked with completing a guided question template while discussing three reading segments related to diagnostic error analysis of the morbidity script for discussion: (A) Cognitive errors, (B) Diagnostic reasoning, or (C) Root cause analysis. Both the jigsaw intervention group and the traditional small group were given the same reading segments and the same allotted time, and their final task was to ‘piece together’ the case based on the reading segments covered. There was no preparation required from the participants for the session. The reading materials were curated to create segments that were covered during both the jigsaw and traditional groups. The overall learning outcome was to: (1) identify three cognitive errors involved in the case, (2) brainstorm a root cause analysis of the event using a fishbone diagram, and (3) solve the major gap in diagnostic reasoning and error analysis that led to the delay in diagnosis. The two faculty facilitators (one for each group) had a training session prior to the workshops in which they reviewed the study flow, the morbidity script, reading segments and written guided template so that they were able to facilitate the discussions in a consistent manner.

#### Jigsaw intervention group

**Figure 1.**
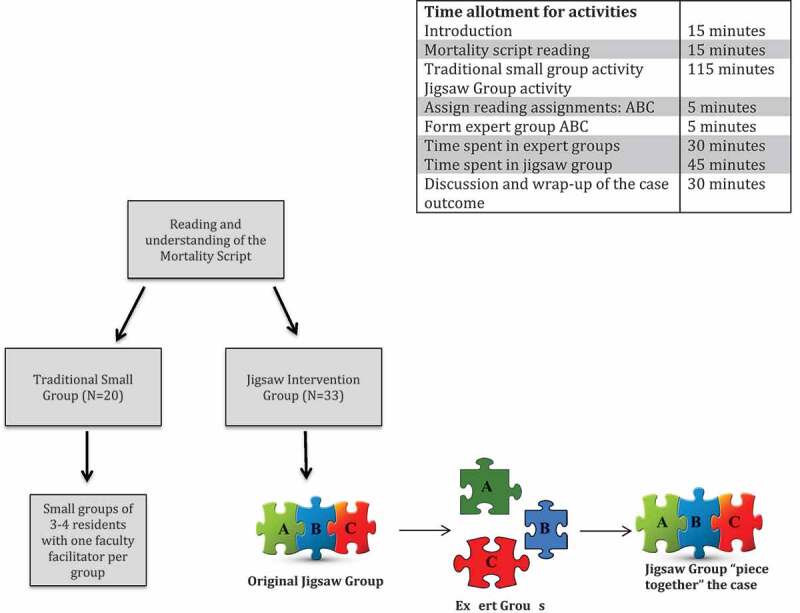
Overall study flow of the workshops with time allotment for each group activity

After reading the morbidity script, participants were organized into jigsaw groups of 3–4 residents each. Each member of the group was assigned to a different reading segment related to diagnostic error analysis: (A) Cognitive errors, (B) Diagnostic reasoning, or (C) Root cause analysis. A faculty facilitator was present to organize and facilitate the group movement and discussions. Group members then joined members of other groups assigned to the same reading piece to form expert groups (i.e., all group A members together, etc.) with the goal of becoming ‘masters’ or experts in the topic by using a guided question template and peer discussion. Eventually, trainees left their expert (A, B, C) groups and returned to their original jigsaw groups (with one-two A members, one B member, one C member) with a task to ‘piece together’ and solve the overall outcome of the case based on their respective expert topics (Figure 1: study flow).

#### Traditional small group

The instructional method used for this group followed the traditional small group exercise format. After reading the morbidity script, participants were then organized into small groups of 3–4 residents each. Each group was assigned a faculty facilitator. During the session all the small groups were assigned the same reading segments related to error analysis: (A) Cognitive errors, (B) Diagnostic reasoning, or (C) Root cause analysis. They were given the same allotted time as the jigsaw groups and were also tasked to ‘piece together’ and solve the overall outcome of the case based on the reading materials covered. The role of the facilitator was to guide the discussion using the written guided template.

#### Data collection and analyses

All test scores were expressed as percentage grade from the faculty facilitators. Overall medical knowledge related to diagnostic errors was assessed using a 10-question multiple choice question quiz. The same quiz was administered to the same cohort of residents one year after intervention to assess for knowledge retention. A 2 × 3 repeated measures analysis of variance (ANOVA) was performed using the following variables: two levels of instructional method (jigsaw and traditional small group) and three levels of time of testing as the repeated measure: before the workshop (T1), after the workshop (T2), and one year follow-up (T3).

The participants also completed a post workshop survey that included yes/no questions regarding whether the workshop content was helpful, what they’ve learned will impact future practice and the amount of time spent on the workshop was worth it. The surveys were administered immediately following the sessions and the results were compared between the two instructional methods groups using a chi-squared test.

## Results

### Test scores

**Figure 2.**
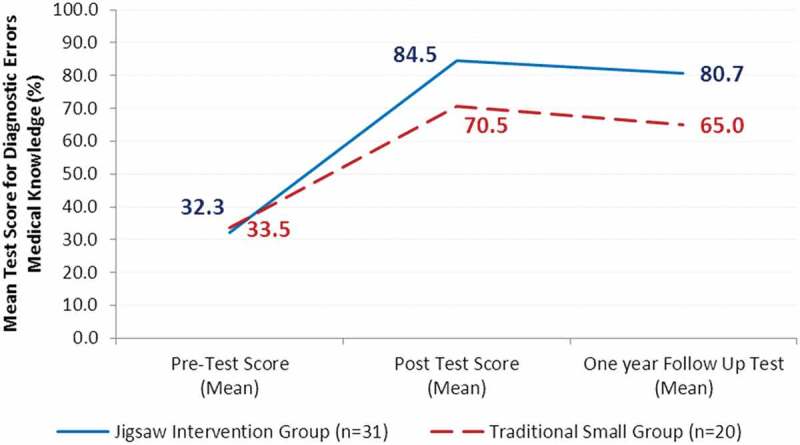
Comparison of medical knowledge of diagnostic errors mean score change from pre-test to post-test and one-year follow-up by instructional method (jigsaw intervention versus traditional small group)

A 2 × 3 repeated measures ANOVA was used to explore the difference between the jigsaw intervention and traditional small groups with respects to pre-test, post-test, and one-year follow-up scores. There was a significant main effect of the instructional method (*F*[1, 49] = 17.9, *p* = .001) as well as of the time of testing variable (*F*[2, 98] = 517.4, *p* = .001) on the overall diagnostic error test scores. There was also a statistically significant interaction between instructional method (jigsaw and traditional small group) and time of testing (T1, T2, and T3) on the test scores (*F*(2, 98) = 17.2, *p* = .001). These results are illustrated in Figure 2 which shows that both the jigsaw intervention group and traditional small group performed better on the post-test compared to the pre-test on medical knowledge of diagnostic errors scores. The jigsaw intervention group had an overall greater improvement in post-test scores. Additionally, although the one-year follow-up test scores decreased for both the jigsaw intervention group and traditional small group compared to the post-test scores, the jigsaw intervention group scored higher than the traditional small group. Post hoc tests showed that the differences in the test scores between the jigsaw and traditional small groups were significant on the post-test (*t*[49] = 4.7, *p* = .001) and on the one-year follow-up test (*t*[49] = 5.3, *p* = .001).

### Survey results

All workshop participants (100%) indicated that the workshop content was helpful. A higher percentage of the participants in the jigsaw intervention group compared to the traditional small group (91% vs. 9%) reported that what they learned from the workshop session would impact future practice (χ^2^ = 32.1, df = 1, *p* < .001). Similarly, more participants in the jigsaw intervention group compared to the traditional small group (68% vs. 32%) revealed that the time they spent participating in the workshop was worth it (χ^2^ = 5.9, df = 1, *p* = .02).

## Discussion

Clinical problem solving remains the central skill of a successful clinician. To master these skills, diagnostic reasoning education among medical trainees is paramount given the high rates of medical errors. Learning should be done in an environment that encourages resident engagement, enabling the residents to serve as simultaneous learner and teacher. To our knowledge, this is the first study that used the jigsaw method to promote active learning and diagnostic reasoning.

Our findings indicate that compared to a traditional small group learning method, using the jigsaw cooperative learning approach resulted in a greater improvement in medical knowledge related to diagnostic reasoning. This was also accompanied by enhanced learner satisfaction as indicated by post-workshop survey feedback. In addition to being an effective method of teaching, the jigsaw method utilizes peer teaching and hence fewer resources, requiring only one facilitator to oversee multiple groups.

Our study demonstrates that the jigsaw method can be a feasible and replicable teaching approach to actively engage learners in clinical problem solving. Furthermore, the jigsaw method emphasizes learner accountability and peer-to-peer teaching.

Our study has many limitations. We relied on learner perception of satisfaction with each learning method. We did not use a crossover design; therefore we cannot directly make a conclusion about comparison of both teaching methods. The pretest scores amongst learners were similar in both groups; we can therefore presume that learners were at similar levels prior to the learning activity. Based on resident time constraints and academic scheduling, we were only able to include PGY one and two. There was no observable difference between PGY one and two level of performance. This may be due to the fact that the PGY two residents did not receive any such training related to patient safety during their PGY one year.

The present study demonstrates that a jigsaw cooperative learning approach can be an effective instructional method to engage trainees in an interdependent manner to learn core concepts related to clinical problem solving. The jigsaw method emphasizes peer teaching, holds each individual accountable for the learning materials, and uses fewer resources (i.e., faculty facilitators) while also emphasizing the importance of teamwork and cooperative learning. Therefore, it can be utilized in other residency programs where faculty time for teaching is limited. Based on the resident’s positive feedback, we plan to utilize the jigsaw teaching on a regular basis during academic Wednesday learning activities.

## Data Availability

All teaching materials described in this manuscript can be made available as supplementary materials or can be accessed by email at nirvani.goolsarran@stonybrookmedicine.edu.

## Prior online publication

This project has not been previously published.

## Prior abstract/poster presentations

This project was presented at the following meeting as poster/abstract presentations: Society of General Internal Medicine (SGIM) National Meeting, 2017, Alliance of Academic Internal Medicine (AAIM)
